# First-principles study of the ternary effects on the plasticity of $\upgamma$-TiAl crystals

**DOI:** 10.1038/s41598-020-77891-5

**Published:** 2020-12-10

**Authors:** Taegu Lee, Seong-Woong Kim, Ji Young Kim, Won-Seok Ko, Seunghwa Ryu

**Affiliations:** 1 Department of Mechanical Engineering, Korea Advanced Institute of Science and Technology, Daejeon, 34141 Republic of Korea; 2 Titanium Department, Korea Institute of Materials Science, Changwon, 51508 Republic of Korea; 3 Department of Materials Science and Engineering, Seoul National University, Seoul, 08826 Republic of Korea; 4 School of Materials Science and Engineering, University of Ulsan, Ulsan, 44610 Republic of Korea

**Keywords:** Atomistic models, Ceramics

## Abstract

We studied the effects of important ternary elements, such as Cr, Nb, and V, on the plasticity of $\upgamma$-TiAl crystals by calculating the point defect formation energy and the change in the generalized stacking fault energy (GSFE) surface from first-principles calculations. For all three elements, the point defect formation energies of the substitutional defects are lower in the Ti site than in the Al site, which implies that substitution on the Ti site is energetically more stable. We computed the GSFE surfaces with and without a substitutional solute and obtained the ideal critical resolved shear stress (ICRSS) of each partial slip. The change in the GSFE surface indicates that the substitution of Ti with Cr, Nb, or V results in an increase in the yield strength because the ICRSS of the superlattice intrinsic stacking fault (SISF) partial slip increases. Interestingly, we find that Cr substitution on an Al site could occur owing to the small difference between the substitutional defect formation energies of the Ti and Al sites. In that case, the reduction of ICRSSs of the SISF partial slip and twinning would lead to improved twinnability. We discuss the implications of the computational predictions by comparing them with experimental results in the literature.

## Introduction

TiAl alloy crystals are intermetallic compounds in which the alpha phase (Ti_3_Al) and the gamma phase (TiAl) form a laminated structure with special orientation relationships. The TiAl alloy is used primarily in the aircraft industry because of its excellent physical properties, such as low density, high strength (${\sigma }_{\mathrm{YS}}=300-800 \mathrm{MPa}$), and good heat resistance for wide range of temperature up to 800 °C^1,2^. However, wider application of the TiAl alloy has been limited because of a critical drawback: low ductility at room temperature that often leads to catastrophic failure^1,2^. To overcome these limitations, many experimental studies have been performed to reveal the deformation mechanism of the TiAl alloy^3–6^. Moreover, previous researchers tested the feasibility of tuning the mechanical properties by adding a third element, the so called the ternary effect. Because the alpha phase is relatively more brittle owing to its limited slip systems^7–9^, most studies related to ternary effects focused on the relatively ductile gamma phase or duplex structures. Previous studies found that the addition of Nb or V (while keeping the atomic fraction of Al constant) increases the yield strength of the gamma TiAl crystal^10–15^, and more deformation twins form with the addition of Mn^16^. Other studies reported that the addition of Cr or V atoms only improves the ductility of the duplex TiAl structure, while not affecting the ductility of single phase gamma^17,18^. Despite the extensive investigations on the ternary effect, a fundamental understanding is still lacking on how a ternary atom affects the mechanical properties of the TiAl gamma phase.

To complement the experimental observations, a couple of theoretical studies reported the first-principle density functional theory (DFT) calculation results of the stacking fault energy (SFE) of the gamma phase or the interface energy of the lamellar structures^19–21^. The SFE of each can be obtained from $\upgamma =\frac{{E}_{sf}-{E}_{0}}{A}$, where ${E}_{sf}$ and ${E}_{0}$ are the energies of crystals with and without the stacking fault, respectively, and $A$ is the area of the slip plane in which a stacking fault lies. Three different stacking faults may form in the gamma TiAl crystal: superlattice intrinsic stacking faults (SISFs), antiphase boundaries (APBs), and complex stacking faults (CSFs). The generalized stacking fault energy (GSFE) surface refers to the entire two-dimensional map made by arbitrary fault vectors that are not necessarily at a local minimum, which offers a more complete picture on the slip system. Moreover, previous research revealed the deformation mechanism of the single nanowire^22^ and the lamellar structure^23,24^ of the binary TiAl alloy. In addition, other studies found that the occupation sites of ternary interstitial sites and the ternary effects on the changes of the lattice constant^25,26^. However, to deepen the understanding of the ternary effect on the mechanical properties, the changes in the stacking faults induced by the ternary atom and the resulting effect on the plastic deformation mechanism need to be predicted. Although the previous calculations show that the changes in the GSFE of the gamma-TiAl-based alloy are due to the ternary substitution^27,28^, the effect of the GSFE change on the plasticity has not been systematically analyzed yet. Furthermore, the relationship between the changes in the GSFE and the mechanical properties by the ternary effects remain to be revealed.

In this study, we investigated the changes in the plastic behaviors of the gamma TiAl crystal upon the addition of Cr, Nb, or V by combining computationally obtained GSFEs and theoretical analyses on the plastic deformation mechanism. First, we calculated the point defect formation energy to determine the preferred site (Ti or Al) for a substitutional ternary atom defect. We then calculated the GSFE surface with and without the substitutional ternary defect. The ideal critical resolved shear stress (ICRSS) of each partial slip was obtained from the maximum slope of a portion of the GSFE surface along the partial Burgers vector. Based on these calculations, we discuss the relationship between the GSFE change and plastic behaviors by considering major plastic deformation mechanisms involving the SISF partial slip, the CSF partial slip, and twinning. We compare the predictions with experimental reports on the ternary effect, such as changes in the yield strength and ductility.

This paper is organized as follows: In Section 2-A, we describe the slip system of the gamma phase in detail and explain the relationship between the GSFE and deformation mechanisms. In addition, we describe the DFT calculation method in Section 2-B. In Section 3, we show the calculation results of the point defect formation energy and the GSFE. We present the ICRSS of each partial dislocation with and without ternary atoms and discuss the ternary effect on the mechanical properties, such as yield strength and ductility. We summarize the results and discuss the outlook in the final section.

## Theoretical background and methodology

### Slip system and deformation mechanism of the gamma TiAl crystals

The gamma phase has an L1_0_ intermetallic structure that is similar to that of the face-centered cubic (FCC) crystal, except that the lattice constant along the *c*-axis (represented as the $[001]$ direction in this study) is approximately two percent longer than the lattice constant along other axes, and two different atomic species are stacked alternately along the *c*-axis. In this study, we adopt the modified Miller indices, which are often used to express the directions and planes in the L1_0_ crystal^29^. Similar to FCC crystals, slip in L1_0_ crystals occurs in the {111) close packed plane. However, because of the different GSFE surface (e.g., three different stacking faults, including SISF, APB, and CSF, may form in the L1_0_ intermetallic structure), the plastic deformation mechanism differs significantly from that of FCC crystals.

**Figure 1:**
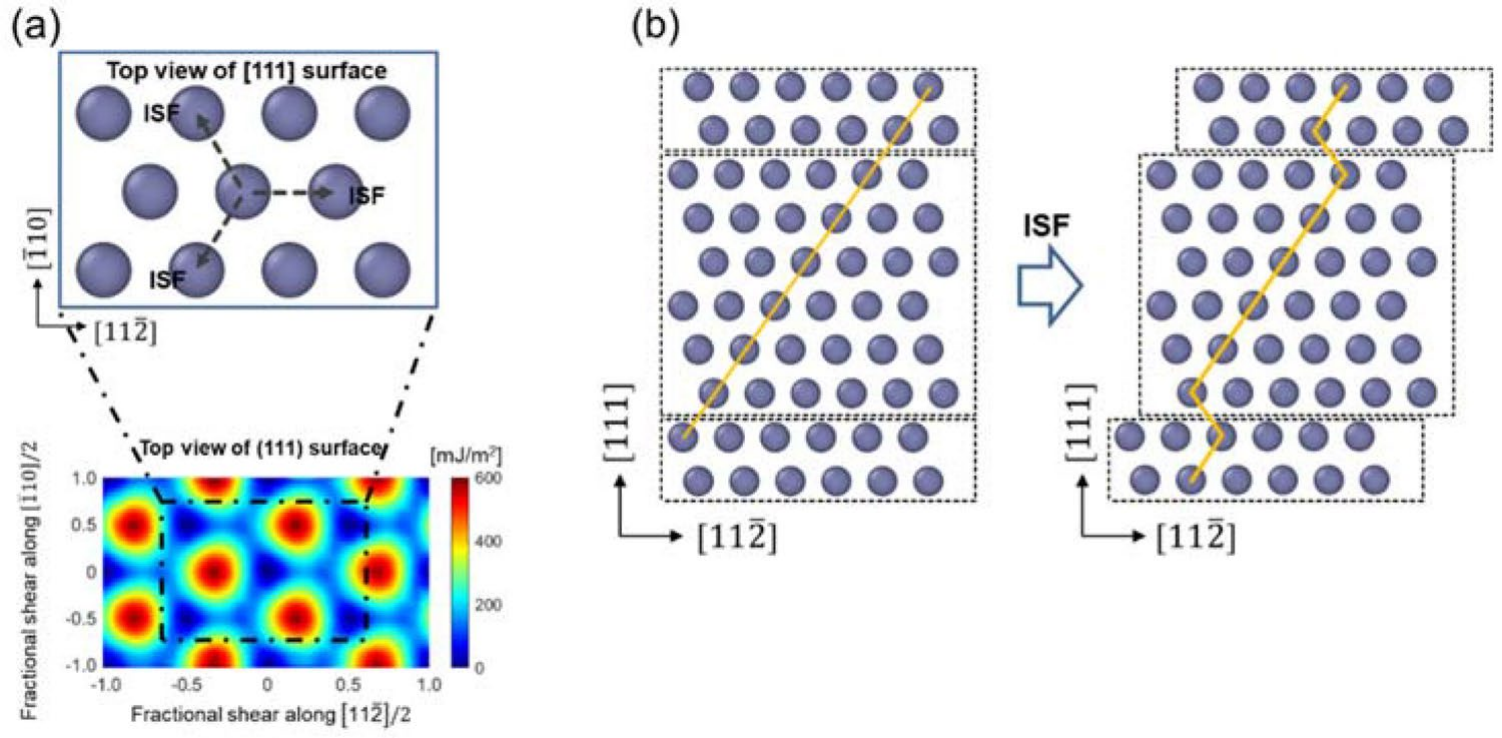
Schematic of the ISF configuration of FCC Al. **(a)** Horizontal view of the GSFE surface and **(b)** vertical view of the ISF partial slip.

**Figure 2:**
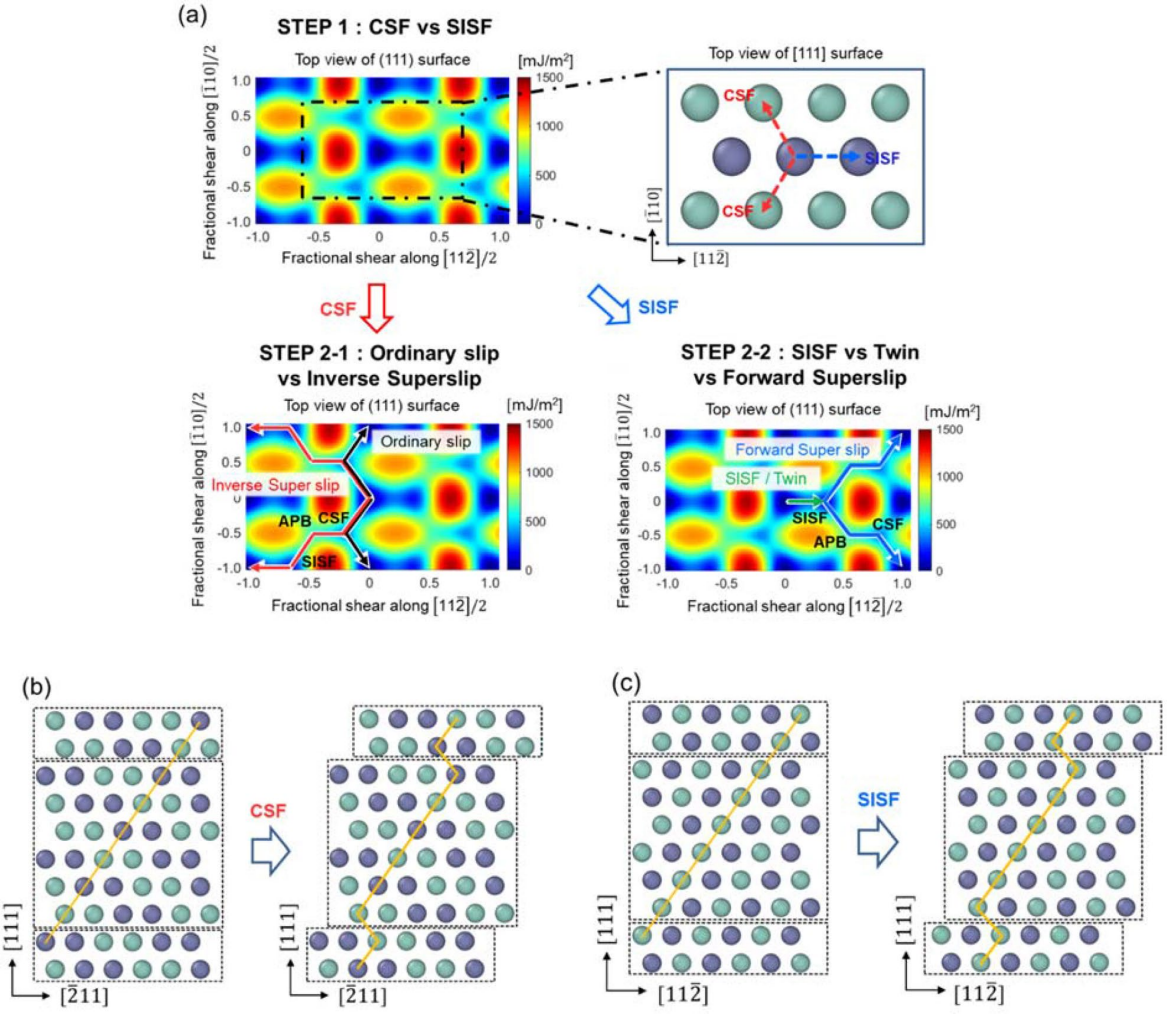
**(a)** Schematic of the configurations of the five deformation mechanisms in gamma TiAl. **(b)** Lateral view of CSF partial slip, and **(c)** lateral view of SISF partial slip.

In the FCC structure, slip on the {111} plane would begin with the 1/6 < 112 > Burgers vector (or the three equivalent directions of 1/6 $\left[11\stackrel{-}{2}\right]$, 1/6 $\left[\stackrel{-}{2}11\right]$, and 1/6 $[1\stackrel{-}{2}1]$), forming the intrinsic stacking fault (ISF), as shown in Fig. 1. Another slip on the same plane along the trailing partial direction would lead to perfect slip, or alternate consecutive slips along the identical direction on the adjacent slip planes would lead to twinning. In contrast, in the L1_0_ structure, the first partial slip would result in either a SISF or CSF, and then subsequent slips would lead to five distinct plastic deformation behaviors. Specifically, the first slip on the {111) plane can occur along two distinguishable directions; slip along the 1/6 < 112] direction leads to the formation of a SISF, while slip along either the 1/6 < 211] or 1/6 < 121] direction would form a CSF, as shown in Fig. 2. When the CSF is initially formed, it can form < 110] ordinary slip by additional slip along 1/6 < 121] (or 1/6 < 211]), which is similar to perfect slip in the FCC lattice. Alternatively, a super slip can form if the additional slip following the CSF occurs to complete the < 011] or 1/2 < 112] super slip, which will be referred to as an inverse super slip in this paper. On the other hand, if a SISF is formed in the first step, the slip process may proceed to form either < 011] or 1/2 < 112] super slip, which will be referred as a forward super slip in this paper. Instead, if the initial SISF is followed by consecutive slips on the adjacent slip planes along the 1/6 < 112] direction, deformation twinning would occur. Alternatively, instead of the deformation twin, multiple SISFs can form on slip planes that are not adjacent to each other. While the perfect dislocation of the FCC structure consists of two partial dislocations, the L1_0_ structure may contain super slip composed of four partial slips or ordinary slip composed of two partial slips^30^ as follows:

1 $$\left[01\stackrel{-}{1}\right]=1/6\left[11\stackrel{-}{2}\right] +\mathrm{SISF}+1/6\left[\stackrel{-}{1}2\stackrel{-}{1}\right]+\mathrm{APB}+1/6\left[11\stackrel{-}{2}\right]+\mathrm{CSF}+1/6\left[\stackrel{-}{1}2\stackrel{-}{1}\right]$$

2 $$1/2\left[11\stackrel{-}{2}\right]=1/6\left[11\stackrel{-}{2}\right] +\mathrm{SISF}+1/6\left[\stackrel{-}{1}2\stackrel{-}{1}\right]+\mathrm{APB}+1/6\left[11\stackrel{-}{2}\right]+\mathrm{CSF}+1/6\left[2\stackrel{-}{11}\right]$$

3 $$1/2\left[\stackrel{-}{1}10\right]=1/6\left[\stackrel{-}{1}2\stackrel{-}{1}\right] +\mathrm{CSF}+1/6\left[\stackrel{-}{2}11\right]$$

In summary, the plastic deformation mechanisms can be categorized into the five different types as follows (see Fig. 2): (i) inverse super slip and (ii) ordinary slip in Step 2–1, and (iii) forward super slip, (iv) deformation twin, and (v) SISFs in Step 2–2. In a previous study^22^, we suggested a framework for predicting the deformation mechanism of a single crystal subjected to uniaxial loading, depending on the loading type (compression or tension) and direction. To determine the type of plastic deformation, the following properties must be considered: the ICRSS, the minimum resolved shear stress required to form the partial slip in the absence of thermal activation, and the Schmid factor, the geometrical factor relating the slip direction and the loading direction. The ICRSS divided by the Schmid factor yields the ideal critical stress required to initiate plastic deformation in a crystal subjected to external loading in the absence of thermal activation. The ICRSS can be obtained from the maximum slope of the GSFE curve along the corresponding partial slip direction. It was shown that the ICRSS has a positive correlation with the critical resolved shear stress and also the critical stress at finite temperatures^31^. For a single crystal subjected to external loading beyond yield, the plastic deformation begins with either the CSF partial slip or the SISF partial slip, which has the lower critical stress of the two. The deformation following the initial partial slip can also be determined by comparing the critical stresses. For instance, if a CSF is formed first, then the slip plane moves in the direction with the lower critical stress between the inverse super slip and the ordinary slip, as shown in Step 2–1. Conversely, if SISF partial slip initially occurs, the plastic deformation proceeds with the mechanism having the lower critical stress, either the forward super slip or the twin in Step 2–2. Based on calculations that show that the CRSS of the twin is always lower than the CRSS of the SISF, we excluded case (v). In our previous study, where full details on the deformation mechanism prediction can be found, we showed that the deformation mechanism predicted based on this framework matches well with molecular dynamics simulations of single crystal nanowires under uniaxial tensile and compressive loading^22^.

Because the plastic deformation behavior is closely related to the mechanical properties of the material, the ICRSS can serve a qualitative indicator for the mechanical properties. The ICRSS of the SISF or the CSF, which occurs first at the onset of plastic deformation, is not only the criterion for dislocation nucleation but also highly related to the ideal shear strength in bulk materials^31^. In particular, the ICRSS of the SISF is lower than that of the CSF (regardless of the ternary atom substitution, as will be shown later), and the change in the yield strength of a polycrystal due to a ternary atom can be inferred from the change in the ICRSS of the SISF. Second, the propensity for deformation twinning is reported to increase the ductility because the twin boundary serves as a barrier for dislocation motion^32–34^. Twin boundaries limit the mean free path of mobile dislocations, and thus plastic deformation becomes more distributed, which enhances the overall ductility. Consequently, it is reasonable to suspect that if the ICRSSs of the SISF and twinning are reduced because of a ternary atom, more twin boundaries would form, and thus the ductility of the material would increase.

### Calculation details of the point defect formation energy and the GSFE

The DFT calculation of the GSFE surface was performed by employing the Vienna Ab initio Simulation Package (VASP) software^35^ with the projector augmented wave (PAW) potentials^36^. The generalized gradient approximation (GGA) as parametrized by Perdew-Burke-Ernzerhof (PBE)^37^ was used to treat electron change and correlation. In the PAW potential for Ti, 3*p* electrons were treated as part of the valence. An energy cutoff of 450 eV and a 5 × 5 × 5 Monkhorst–Pack *k*-point mesh^38^ were used for the formation energy calculations for all supercells considered in the study, and the same energy cutoff and a 9 × 7 × 3 Monkhorst–Pack *k*-point mesh were used for the SFE calculations.

Three different elements (Cr, Nb, and V), which have been widely used in experiments, were chosen as the ternary elements in the present study. In order to determine whether a Ti or an Al site is more stable for the ternary element substitution, we defined the point defect formation energy $\Delta$ at 0 K which is the difference between the formation energies of a ternary alloy and the stoichiometric gamma TiAl as follows:

4 $$\Delta \left({\mathrm{Ti}}_{\mathrm{N}-1}{\mathrm{Al}}_{\mathrm{N}}{\mathrm{X}}_{1}\right)=E\left({\mathrm{Ti}}_{\mathrm{N}-1}{\mathrm{Al}}_{\mathrm{N}}{\mathrm{X}}_{1}\right)-E\left({\mathrm{Ti}}_{\mathrm{N}}{\mathrm{Al}}_{\mathrm{N}}\right)+E\left({\mathrm{Ti}}_{1}^{\mathrm{hcp}}\right)-E\left({\mathrm{X}}_{1}^{\mathrm{bcc}}\right)$$

5 $$\Delta \left({\mathrm{Ti}}_{\mathrm{N}}{\mathrm{Al}}_{\mathrm{N}-1}{\mathrm{X}}_{1}\right)=E\left({\mathrm{Ti}}_{\mathrm{N}}{\mathrm{Al}}_{\mathrm{N}-1}{\mathrm{X}}_{1}\right)-E\left({\mathrm{Ti}}_{\mathrm{N}}{\mathrm{Al}}_{\mathrm{N}}\right)+E\left({\mathrm{Al}}_{1}^{\mathrm{fcc}}\right)-E\left({\mathrm{X}}_{1}^{\mathrm{bcc}}\right)$$

Ti_N_Al_N_ indicates stoichiometric TiAl consisting of *N* atoms for each element, and ${\mathrm{Al}}_{\mathrm{N}-1}$ or ${\mathrm{Ti}}_{\mathrm{N}-1}$ indicates that one atom of the corresponding sublattice site is replaced by an X (Cr, Nb, or V) atom. The energy of a single atom was calculated for a stable structure under the ambient condition (i.e., FCC for Al, HCP for Ti, and BCC for Cr, Nb, and V).

To check possible artifacts from the finite size effect, L1_0_ supercells (x: [$100$], y: [$010$], z: [$001$]) consisting of 32 atoms (ternary element ~ 3 at.%) and 108 atoms (ternary element ~ 1 at.%) were used. Both the unrelaxed and relaxed point defect formation energies of all three species of atoms were lower when the ternary atom occupied a Ti site (The detailed explanation is in the Results and discussion section (Section 3)).

**Figure 3:**
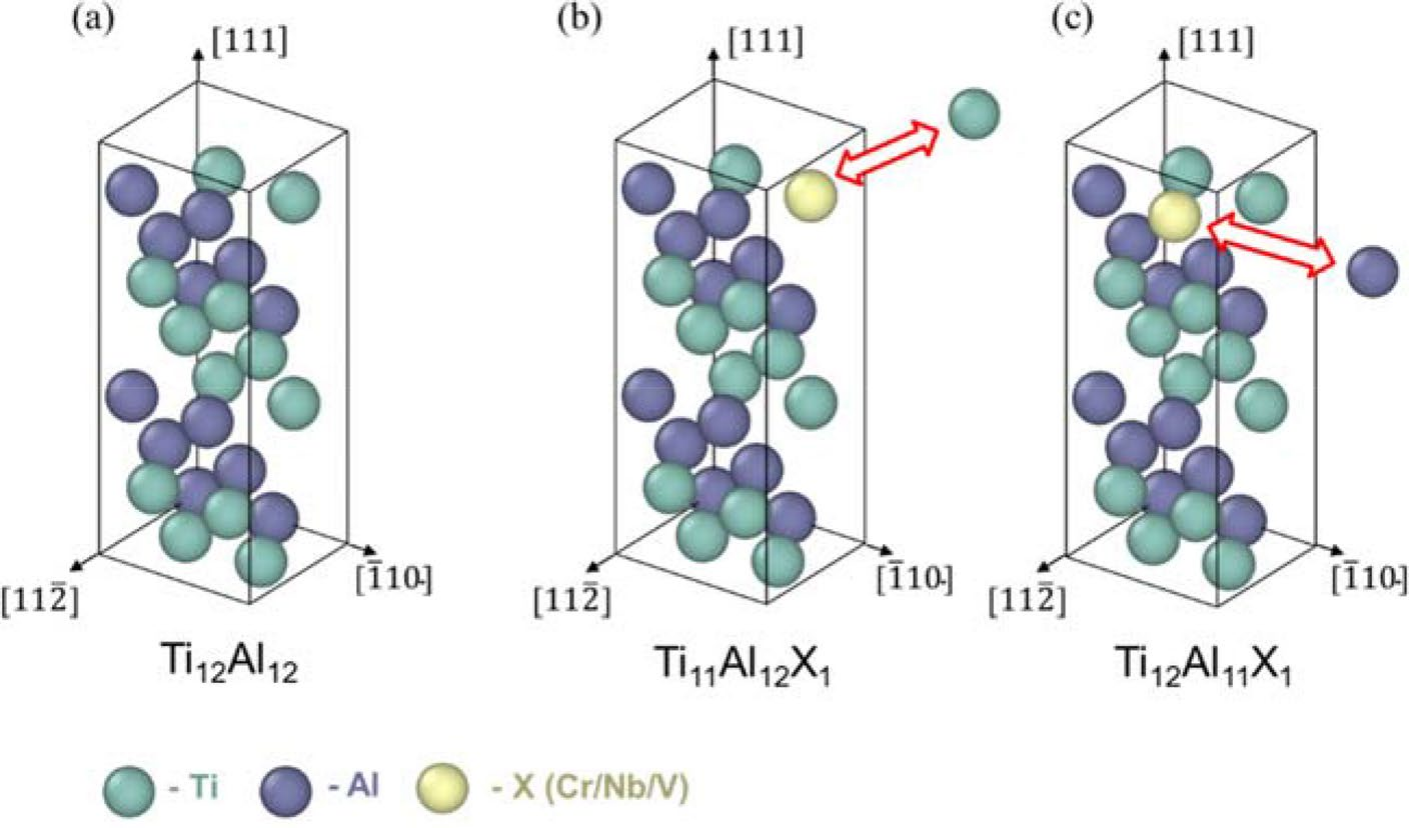
Atomic configurations of supercells for **(a)** stoichiometric gamma TiAl (Ti_12_Al_12_), **(b)** Ti_11_Al_12_X_1_ (~ 4 at.% ternary atom substituted for Ti), and **(c)** Ti_12_Al_11_X_1_ (~ 4 at.% ternary atom substituted for Al).

**Figure 4:**
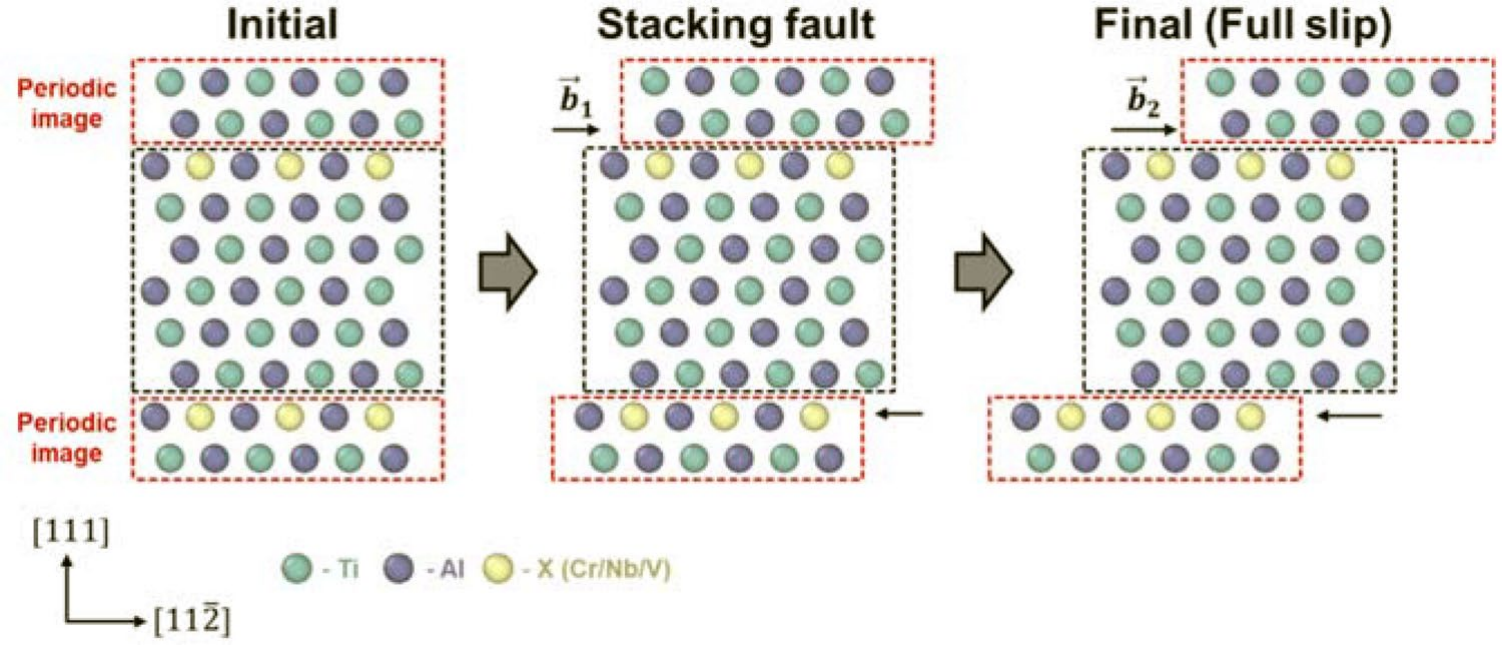
Stacking faults model for GSFE calculation.

Next, the GSFE surface at 0 K was computed with and without the three different ternary elements. Figure 3a shows the L1_0_ supercell (x: [$11\overline{2}$], y: [$\overline{1}10$], z: [$111$]) consisting of 24 atoms that was used to calculate the SFE of the ternary TiAl-based alloys. Owing to the computational cost, smaller cells were used for the SFE calculations than the one used for the formation energy calculations. In addition, the calculation of the formation energy was repeated for a smaller supercell. Despite the size and orientation of the supercell, the relative order between the formation energies for the Ti and Al sites did not change. When the ternary atom was substituted for a Ti atom or an Al atom in the supercell, the atomic percent of the ternary atom was approximately 4 at.%, as shown in Fig. 3b,c. Because the GSFE was obtained by tilting the periodic boundaries while maintaining the real positions of atoms, as depicted in Fig. 4, the topmost Ti or Al atom was substituted with the ternary atom to locate the substitutional defect adjacent to the designated slip plane. Given the three vectors of the initial supercell periodic boundaries (namely, $\overrightarrow{{a}_{1}}$,$\overrightarrow{{a}_{2}}, \mathrm{and }\overrightarrow{{a}_{3}}$, where $\overrightarrow{{a}_{1}}$ and $\overrightarrow{{a}_{2}}$ are parallel to the slip plane), the total energy of the system was computed as $\overrightarrow{{a}_{3}}$ was changed following $\overrightarrow{{a}_{3}}\mathrm{^{\prime}}=n\overrightarrow{{a}_{3}}+\overrightarrow{b}$, where the relative displacement, $\overrightarrow{b}=l\overrightarrow{{a}_{1}}+m\overrightarrow{{a}_{2}}$, was intended to scan the entire slip vector on the slip plane, and $n\overrightarrow{{a}_{3}}$ was adjusted to obtain relaxed GSFEs at a given slip vector, $\overrightarrow{b}$. In detail, $\overrightarrow{{a}_{1}}$,$\overrightarrow{{a}_{2}}$, and $\overrightarrow{{a}_{3}}$ were 4.953, 5.636, and 14 Å, respectively. Values of $l$, $\mathrm{m}$, and $n$ were $\left(0,\frac{1}{20},\frac{2}{20},\dots , 1\right)$, $\left(0,\frac{1}{12},\frac{2}{12},\dots ,1\right)$, and $\left(\frac{23}{28},\frac{25}{28},\dots ,\frac{33}{28}\right)$, respectively. In the other words, the relaxed GSFE surface was obtained by computing the energies for different values (21, 13, and 6) of $l,\mathrm{ m},\mathrm{ and n}$, respectively (in total, 1638 cases).

**Figure 5:**
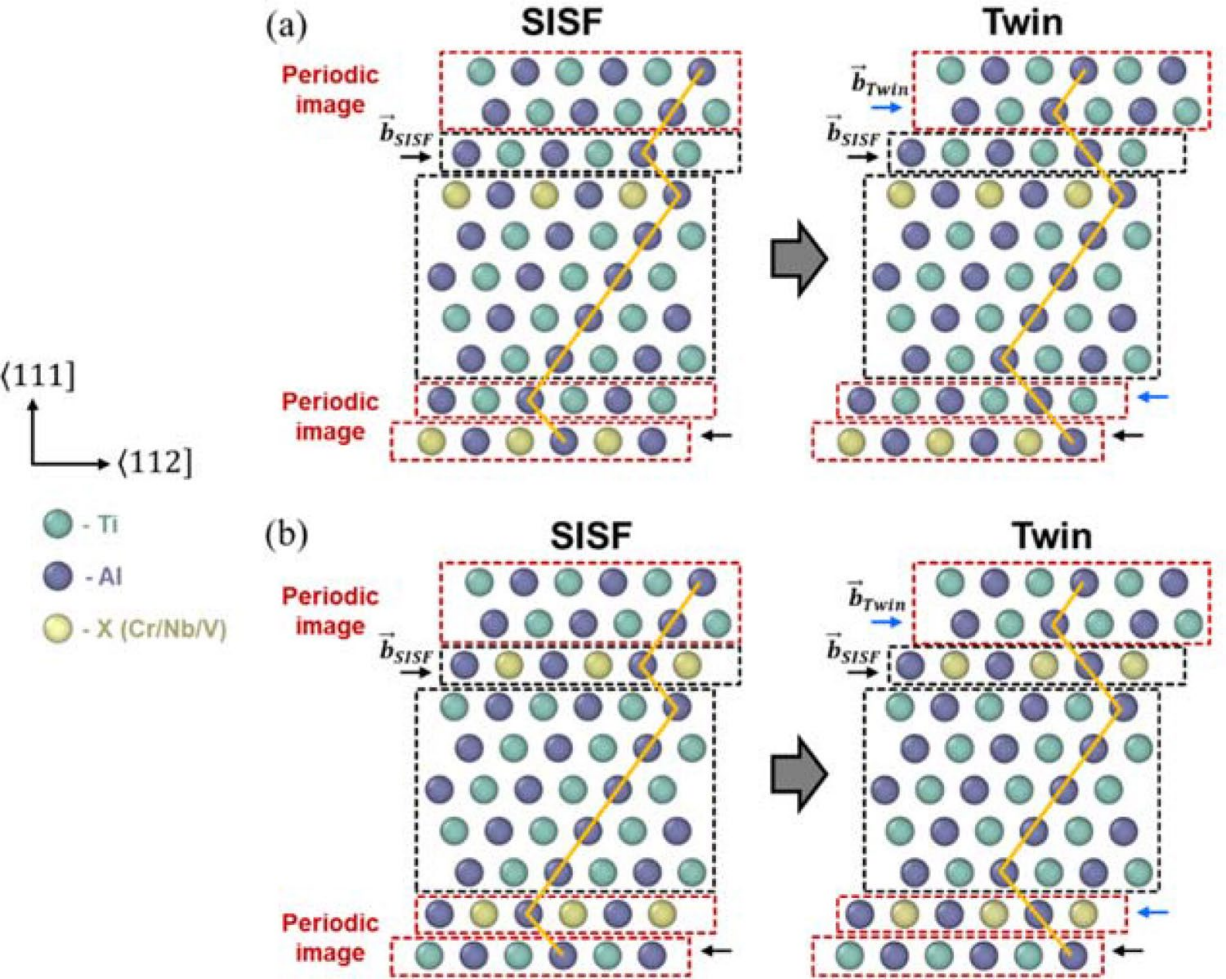
Two possible models for twinning: **(a)** the ternary alloying element X (Cr, Nb, or V) is located in a single slip plane and **(b)** the ternary alloying element X is located between both slip planes.

Afterward, starting from a simulation cell possessing a SISF, the GSFE curve was obtained by considering the slip along the slip plane adjacent to the SISF to calculate the ICRSS of twinning. For the twinning, two scenarios were considered, as visualized in Fig. 5a,b. The first scenario considered the case in which the ternary atom was located next to the initial slip plane but apart from the subsequent slip plane (Fig. 5a), while the second scenario assumed that the ternary atom was located between two consecutive slip planes (Fig. 5b). When predicting the deformation mechanism, the ICRSS of twinning was set to the smaller maximum slope between the two scenarios.

## Results and discussion of the first-principles calculations of the point defect formation energies and the GSFE surfaces

Table 1 lists the point defect formation energies obtained from the first-principles DFT calculations. The point defect formation energies obtained from the supercells used for the GSFE are presented in the Supplementary Information (Fig. S2 and Tables S1-S2). The results reveal that Cr, Nb, and V defects preferentially substitute on the Ti site over the Al site as demonstrated by the lower point defect formation energies of substitutional point defects for the Ti site compared with those for the Al site. The difference between the substitutional energies for Ti and Al sites are the largest for Nb and the smallest for Cr. For Nb and V, the difference between the point defect formation energies for the Ti and Al sites is significantly larger than the thermal energy at room temperature (~ 0.026 eV). Hence, unless a ternary gamma TiAl alloy with Nb or V is quenched at an extremely fast cooling rate, the ternary element is likely to occupy the Ti sites. In contrast, the point defect formation energy difference for Cr substitutional defects is comparable to the thermal energy, and a non-negligible fraction of Cr would occupy some portion of the Al sites. The formation energy calculation is consistent with previous ALCHEMI (atom location by channeling-enhanced microanalysis) experiments on a single gamma phase indicating that V occupies only the Ti sites and Cr occupies both the Ti and Al sites^17,18^.

**Table 1:** Relaxed (without the parentheses) and unrelaxed (in the parentheses) point defect formation energies of each ternary alloy considering the substitution of a Ti or an Al site by an alloying element X (Cr, Nb, or V). Results for supercells with 32 and 108 atoms are presented.

Point defect formation energy (eV)	Cr	Nb	V
${\mathrm{Ti}}_{15}{\mathrm{Al}}_{16}{\mathrm{X}}_{1}$	1.015 (1.190)	0.191 (0.196)	0.501 (0.567)
${\mathrm{Ti}}_{16}{\mathrm{Al}}_{15}{\mathrm{X}}_{1}$	1.076 (1.422)	0.904 (1.014)	0.948 (1.131)
${\mathrm{Ti}}_{53}{\mathrm{Al}}_{54}{\mathrm{X}}_{1}$	0.984 (1.179)	0.182 (0.192)	0.497 (0.562)
${\mathrm{Ti}}_{54}{\mathrm{Al}}_{53}{\mathrm{X}}_{1}$	1.019 (1.309)	0.847 (0.939)	0.884 (1.070)

**Figure 6:**
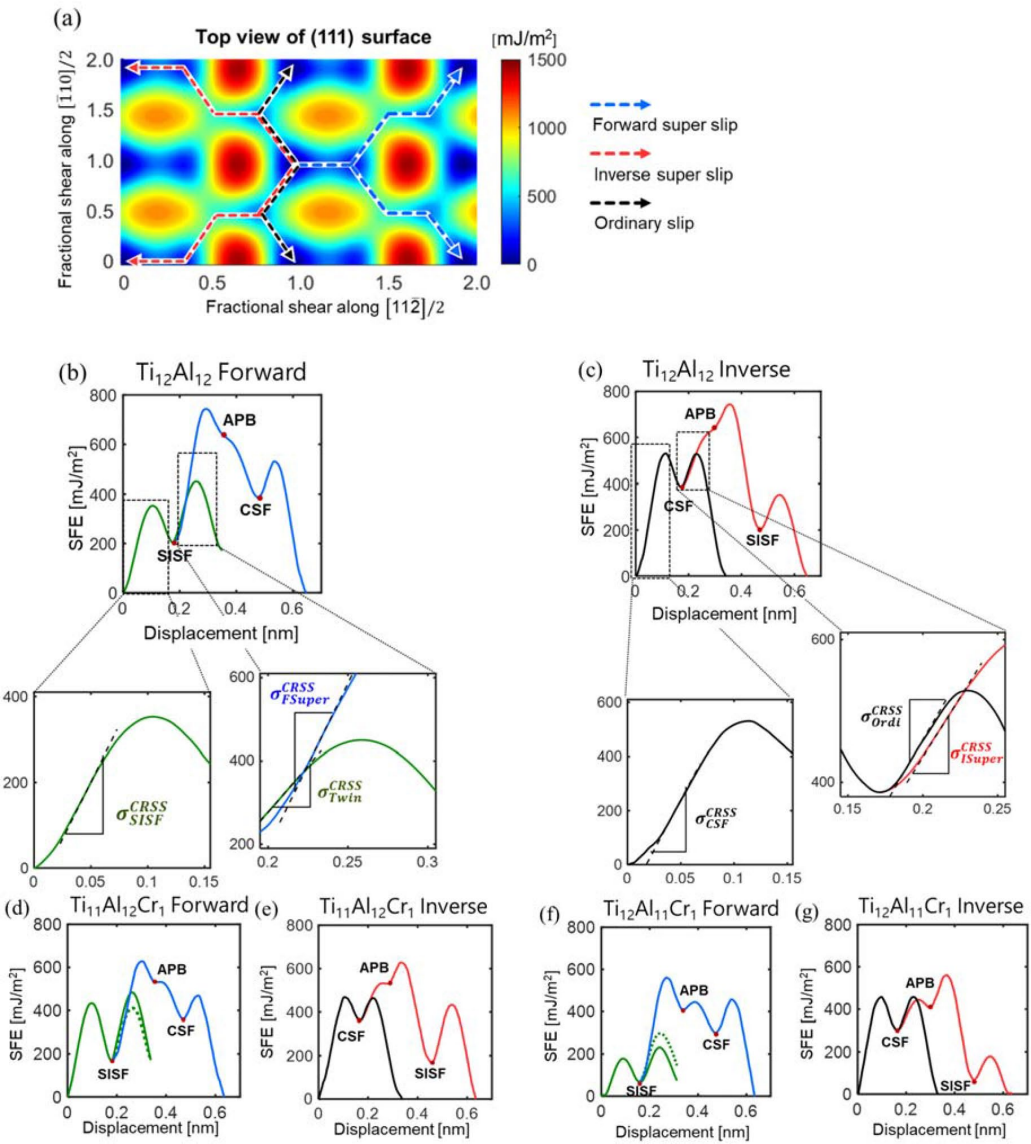
**(a)** GSFE surface of stoichiometric gamma TiAl (Ti_12_Al_12_). GSFE curves for **(b)** forward super slip and twinning (the maximum slope of the GSFE curve is the critical resolved shear stress) and **(c)** inverse super slip and ordinary slip in stoichiometric gamma TiAl. GSFE curves for **(d)** forward super slip and twinning, **(e)** inverse super slip and ordinary slip, **(f) **forward super slip and twinning, and **(g)** inverse super slip and ordinary slip in Ti_12_Al_11_Cr_1_ (the blue line is forward super slip, the red line is inverse super slip, the black line is ordinary slip, and the green line is twinning).

The calculated GSFE surface is shown in Fig. 6a. The blue dashed line indicates the slip path for forward super slip, the red dashed line indicates the slip path for inverse super slip, and the black dashed line indicates the slip path for ordinary slip. By taking the GSFEs along the paths and augmenting the GSFE curve for twinning, a set of GSFE curves can be extracted, as shown in Fig. 6b,c. Table 2 summarizes the SFE values for the SISFs, APBs, and CSFs obtained for each ternary alloy. The GSFE curves for when a Cr atom occupies either a Ti or an Al site are shown in Figs. 6 (d)-(g). The GSFE curves for Nb and V substitution are depicted in Fig. S3 in the Supplementary Information. Here, the curve from the first twinning scenario appears as a solid line, while the curve from the second twin scenario is shown as a dotted line. The ICRSS was obtained for various deformation mechanisms by computing the maximum slopes for three parts (SISF formation, twinning, and forward super slip) of the GSFE curves starting from the SISF formation, as well as maximum slopes for three parts (CSF formation, ordinary slip, and inverse super slip) of the GSFE curves starting from the CSF formation. Table 3 summarizes the ICRSSs for all cases and the relative changes in the ICRSSs of ternary alloys (for both Ti and Al site substitution) with respect to the values of the stoichiometric gamma TiAl alloy. For all ternary elements, two remarkable common trends are present. First, the substitution of a ternary atom in a Ti site increases the ICRSS of the SISF partial slip. Second, the substitution of a ternary atom in an Al site significantly decreases the ICRSS of the SISF and twinning. Out of six total cases (three substitution atoms in the Ti or Al site), only four cases were consider by excluding two cases (Nb in Al sites, V in Al sites) that are not likely to occur in realistic experiments on gamma TiAl crystals ^17,18^.

**Table 2:** Calculated stacking fault energies of each alloy. Results for supercells with 24 atoms are presented. Corresponding atomic configurations are presented in Fig. 3.

SFE (mJ/m^2^)	SISF	APB	CSF
Ti_12_Al_12_	203	638	385
Ti_11_Al_12_Cr_1_	166	530	360
Ti_11_Al_12_Nb_1_	239	636	459
Ti_11_Al_12_V_1_	199	554	376
Ti_12_Al_11_Cr_1_	65.7	407	301
Ti_12_Al_11_Nb_1_	74.7	266	217
Ti_12_Al_11_V_1_	41.4	320	246

**Table 3:** ICRSS and the relative change compared with stoichiometric gamma TiAl.

ICRSS (GPa) (%)	ORI → SISF (Forward dir.)			ORI → CSF (Inverse dir.)		
	${\sigma }_{SISF}^{CRSS}$	${\sigma }_{Twin}^{CRSS}$	${\sigma }_{FSuper}^{CRSS}$	${\sigma }_{CSF}^{CRSS}$	${\sigma }_{Ordi}^{CRSS}$	${\sigma }_{ISuper}^{CRSS}$
Ti_12_Al_12_	5.20	4.71	8.07	8.45	4.12	3.09
Ti_11_Al_12_Cr_1_	6.58	5.24	6.20	7.52	3.21	3.11
	26.7%	11.2%	− 23.2%	− 11.0%	− 22.2%	0.59%
Ti_11_Al_12_Nb_1_	5.72	4.41	7.96	8.11	3.31	3.02
	10.1%	− 6.42%	− 1.37%	− 3.95%	− 19.8%	− 2.16%
Ti_11_Al_12_V_1_	6.25	4.65	8.34	8.07	4.03	3.09
	20.3%	− 1.23%	3.35%	− 4.49%	− 2.18%	0.01%
Ti_12_Al_11_Cr_1_	3.60	3.18	7.35	8.33	3.39	2.57
	− 30.8%	− 32.4%	− 8.96%	− 1.43%	− 17.7%	− 16.9%
Ti_12_Al_11_Nb_1_	2.75	1.91	6.31	8.83	3.18	3.03
	− 47.2%	− 59.5%	− 21.9%	4.50%	− 23.0%	− 2.09%
Ti_12_Al_11_V_1_	3.47	3.08	7.40	8.12	3.63	2.66
	− 33.2%	− 34.5%	− 8.31%	− 3.90%	− 11.9%	− 14.1%

As mentioned in the previous section (Section 2-B), the ICRSS of the SISF is notably smaller than the ICRSS of the CSF for all the gamma TiAl alloys considered in the study. Hence, the ICRSS of the SISF is likely related to the yield strength of polycrystalline gamma TiAl. Because ternary atoms would occupy the Ti sites and the ICRSS of SISF slip increases in the presence of ternary atoms (as depicted in Table 3 and Fig. 6), our combined computational and theoretical analysis indicate that the yield strengths of ternary alloys are likely to increase. Our prediction is consistent with previous experimental studies^10–14^ that report strengthening due to the ternary atom addition. We note that the SISF energy of the ternary alloy is similar to the SISF energy of the stoichiometric gamma TiAl alloy (Fig. 6 and Table 2); therefore, the results confirm that the energy barrier and the maximum slope of the SISF are more relevant criteria than the specific value of the SISF energy.

**Figure 7:**
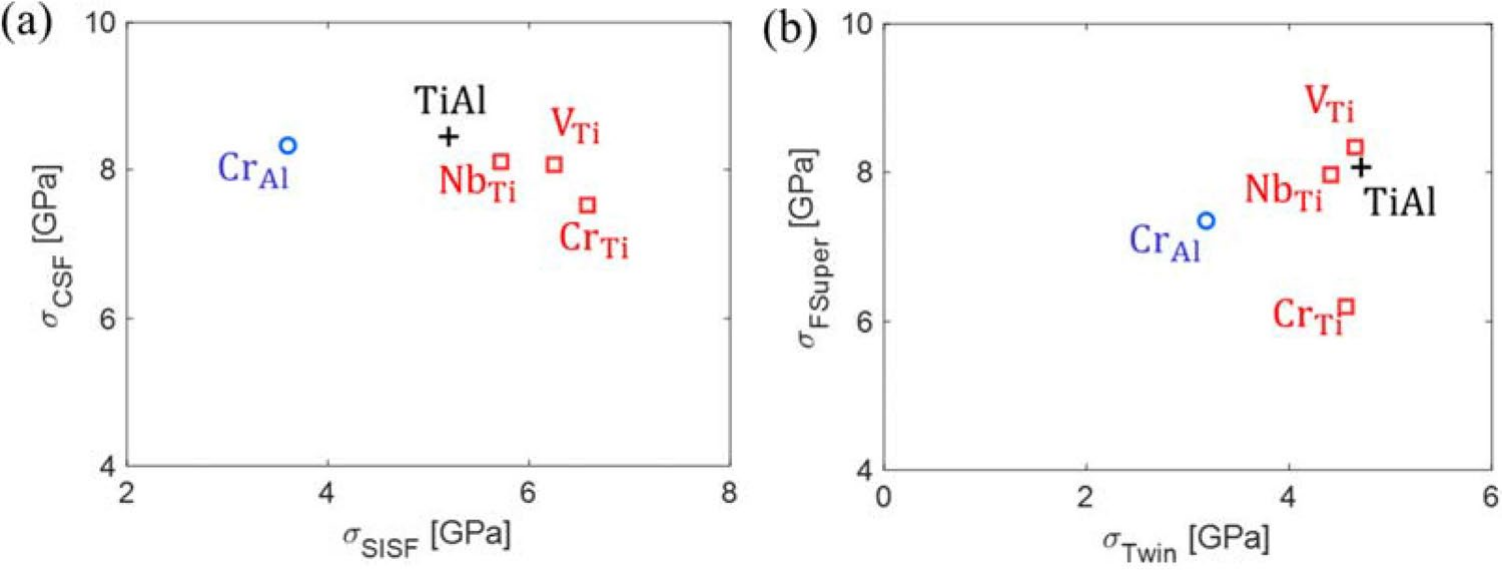
**(a)** ICRSSs of the SISFs and CSFs. **(b)** ICRSSs of twinning and forward super slip (X_Ti_: substitution in a Ti site, X_Al_: substitution in an Al site).

In contrast, for substitutional defect formation in Al sites, the ICRSS of SISF slip decreased by 30–47%, and the ICRSS of twinning decreased by 32–60%, as presented in Table 3 and Fig. 7. In addition, the ICRSS of forward super slip also decreases. For the quantitative analysis of the propensity for deformation twinning, the twinnability factor (T.F.) is defined as follows:

6 $$\mathrm{T}.\mathrm{F}. = \frac{{\sigma }_{CSF}^{CRSS}}{{\sigma }_{SISF}^{CRSS}}\times \frac{{\sigma }_{FSuper}^{CRSS}}{{\sigma }_{Twin}^{CRSS}}$$

**Figure 8:**
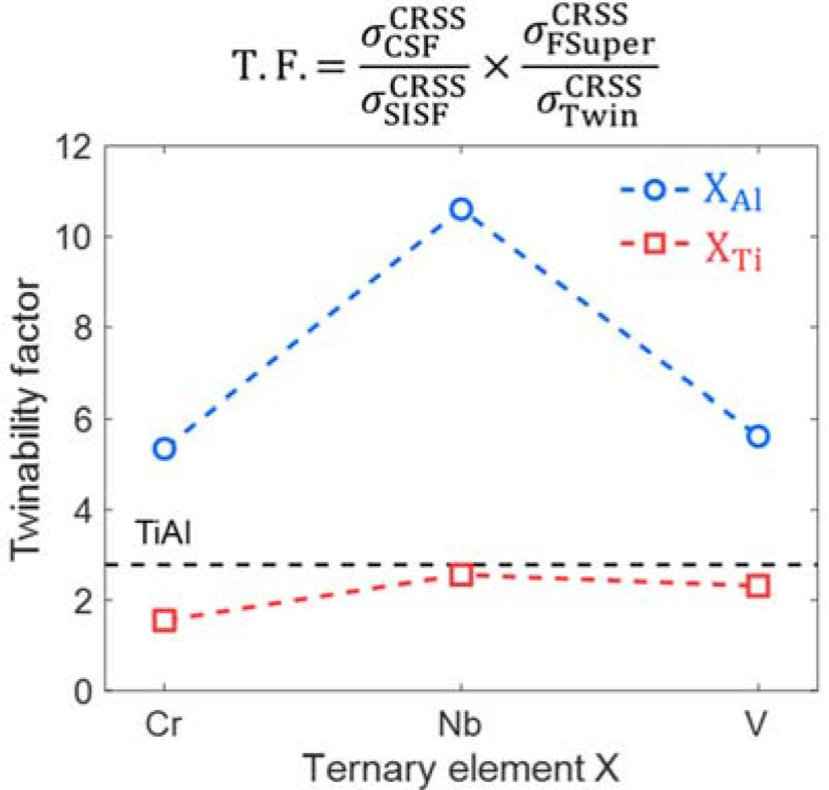
Calculated twinnability factor of each ternary alloy considering the substitution of a Ti (X_Ti_) or Al (X_Al_) site by an alloying element X (Cr, Nb, or V).

This is the product of the ratio of the ICRSS of the CSF (${\sigma }_{CSF}^{CRSS}$) and the SISF (${\sigma }_{SISF}^{CRSS}$) with the ratio of the ICRSS of forward super slip (${\sigma }_{FSuper}^{CRSS}$) and twinning (${\sigma }_{Twin}^{CRSS}$). If this factor is high, twinning is generally preferred over dislocation slip. Figure 8 shows the effect of the substitution of a Ti or an Al site by an alloying element (Cr, Nb, or V) on the twinnability factor. The substitution of the Ti site exhibits only a marginal difference in the twinnability factor compared to the stoichiometry TiAl alloy, while the substitution of the Al site exhibits a more significant difference. It was found that ternary substitution in an Al site would result in an increase in the twinnability factor. However, because only a small portion of the Cr is expected to occupy the Al sites instead of the Ti sites, it is not straightforward to discuss the effect of the Cr on the plastic deformation mechanism of gamma TiAl crystals.

**Figure 9:**
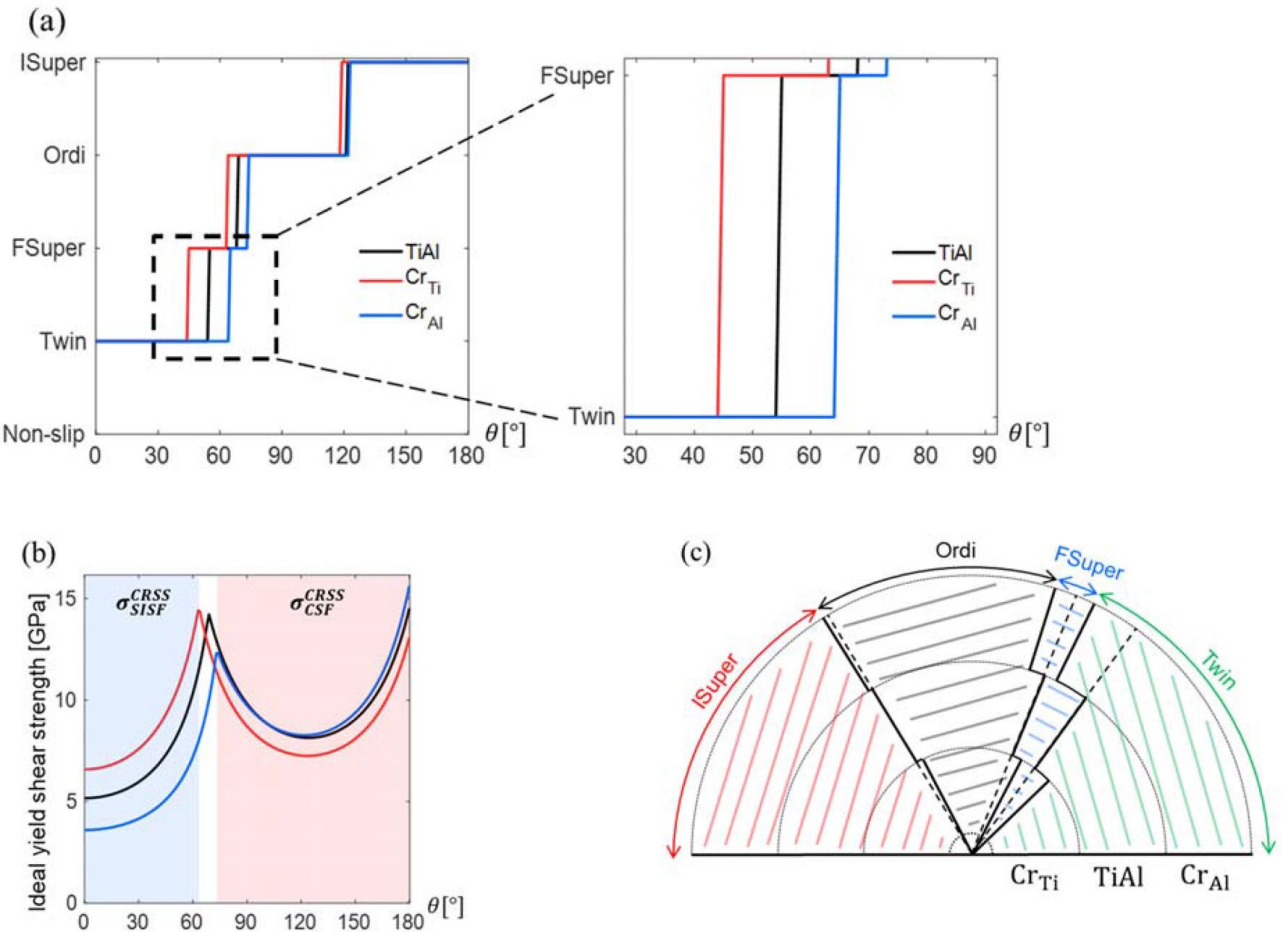
**(a)** Predicted deformation mechanism. **(b)** Ideal yield shear strength (the blue and red shaded regions represent SISF and CSF, respectively). **(c)** Predicted deformation mechanism diagram.

In addition, to visualize and confirm the ternary effect, the ideal plastic deformation mechanism was predicted from the ideal shear strength comparison method^22^ for Cr addition, which occupies both the Ti and the Al sites. First, the [$11\stackrel{-}{2}$] vector was set as *x*-axis on the ($111$) slip plane, and the shear stress applied on the slip plane from 0 to 180° counterclockwise to the *x*-axis was examined. The predicted deformation behaviors and the ideal yield shear strength of the stoichiometric L1_0_ TiAl and the ternary TiAl-based alloys are presented in Fig. 9a,b, respectively. In Fig. 9b, the blue region represents conditions for dominant SISF formation, while the red region represents conditions for dominant CSF slip, and the intermediate white region represents transient conditions affected by the ternary elements. As shown in Fig. 9b, the substitution of the Ti sites by Cr atoms facilitates the forward super slip with an increased yield shear strength. Conversely, the substitution of the Al sites by Cr atoms facilitates twinning with a decreased yield shear strength. A schematic that summarizes the expected deformation behavior is shown in Fig. 9c.

To summarize the discussion, the GSFE calculations show that a ternary atom substituted for Ti increases the yield strength, while a ternary atom substituted for Al increases the ductility. In other words, the GSFE change pattern is depending on the Al content in TiAl and the kind of the ternary atom affects the magnitude of GSFE changes. These results are consistent with previous experimental papers.^13,14^ Our prediction on the yield strength due to a ternary atom occupying a Ti site is consistent with existing experimental studies^10–14^ that report a strengthening effect induced by ternary alloying elements. In contrast, from GSFE surface analysis, it was found that all three ternary atoms in the Ti site would have a negligible effect on the ductility, while a ternary atom in the Al site may improve the ductility. However, because the portion of ternary atoms in the Al sites is significantly smaller than the ternary atoms in the Ti sites, the ternary effect on ductility in gamma TiAl phase is expected to be insignificant. Our results are consistent with the existing experimental studies^17,18^, where the ductility of pure gamma TiAl alloy does not change significantly upon the addition of ternary alloying elements. Our study indicates that, to explain the ductility increase in the duplex structure by ternary atoms, it is necessary to consider not only the gamma phase but also the interfacial effects between the gamma phase and the alpha phase as well as microstructural effects, such as grain size and lamellar size and spacing.

## Conclusions

We calculated the point defect formation energy and the GSFE of TiAl-based ternary alloys to analyze the changes in the mechanical properties by adding a third element, such as Cr, Nb, or V, to the gamma TiAl. First, from the point defect formation energy calculation, we found that all ternary substitutional atoms are more stable in the Ti site than in the Al site. In the case of Cr, the point defect formation energy difference between the Ti and Al sites is comparable to the thermal energy at ambient conditions. Our calculations are consistent with experimental reports that Nb and V substitutional defects occupy Ti sites, while Cr substitution is found in both Ti and Al sites. Next, we calculated the ternary-atom-induced change in the GSFE and the ICRSS for each partial dislocation via the maximum slope of the GSFE curve. The results suggest that ternary atoms occupying Ti sites increases the yield strength of the alloy because of the increase in the ICRSS of the SISF. In contrast, Cr substitution in Al sites decreases the ICRSS of the SISF and twin, increasing the propensity for twinning. However, the effect on the ductility of the gamma TiAl alloy is limited because a significantly larger portion of the Cr atoms would occupy Ti sites.

In summary, we theoretically explain the previous experimental studies reporting that the yield strength increases due to substitutional defects, while the effect on the ductility of the single phase gamma is limited. Our study indicates that further study is necessary to deepen our understanding on the ductility improvement of the duplex structure. We note that the framework used in the present study can be applied to investigate the ternary effect on a variety of intermetallic systems.
